# Novel regional longitudinal strain by speckle tracking to detect significant coronary artery disease in patients admitted to the emergency department for chest pain suggestive of acute coronary syndrome

**DOI:** 10.1007/s12574-022-00568-7

**Published:** 2022-03-15

**Authors:** Ingvild Billehaug Norum, Jan Erik Otterstad, Vidar Ruddox, Bjørn Bendz, Thor Edvardsen

**Affiliations:** 1 Department of Cardiology, Vestfold Hospital Trust, P. O Box 2168, 3103 Tønsberg, Norway; 2 Faculty of Medicine, University of Oslo, P.O Box 1078, 0316 Oslo, Norway; 3 Department of Cardiology, Division Rikshospitalet, Oslo University Hospital, P.O Box 4950, 0424 Oslo, Norway

**Keywords:** Regional longitudinal strain, Chest pain, Coronary artery disease, Regional myocardial function

## Abstract

**Background:**

Global longitudinal strain has shown variable results in detecting ischemia in patients admitted to the emergency department with chest pain, but without other clear evidence of coronary artery disease (CAD). Our aim was to investigate whether assessment of regional longitudinal myocardial function could assist in detecting significant CAD in these patients.

**Methods:**

Clinical evaluation, electrocardiogram, echocardiogram and troponin T were evaluated in 126 patients admitted with chest pain. A subsequent invasive coronary angiography divided patients into two groups: significant CAD (CAD+) or non-significant CAD (CAD−). Global and regional myocardial function were evaluated by speckle tracking echocardiography. Regional longitudinal strain was defined as the highest longitudinal strain values in four adjacent left ventricular segments and termed 4AS.

**Results:**

CAD+ was found in 37 patients (29%) of which 51% had elevated troponin. Mean 4AS was − 13.1% (± 3.5) in the CAD+ and − 15.2% (± 2.7) (*p* = 0.002) in the CAD− group. Predictors for CAD+ were age [OR 1.06 (1.01–1.11, *p* = 0.026)], smoking [OR 3.39 (1.21–9.51, *p* = 0.020)], troponin [OR 3.32 (1.28–8.60, *p* = 0.014)) and 4AS (OR 1.24 (1.05–1.46, *p* = 0.010)]. A cutoff for 4AS of > − 15% showed the best diagnostic performance with event-reclassification of 0.41 (*p* < 0.001), non-event-reclassification of − 0.34 (*p* < 0.001) and net reclassification improvement 0.07 (*p* = 0.60).

**Conclusion:**

Decreased myocardial function in four adjacent LV segments assessed by strain has the potential to detect significant CAD in patients admitted with chest pain and negative/slightly elevated initial troponin.

**Trial registration:** Current Research information system in Norway (CRISTIN). Id: 555249.

**Supplementary Information:**

The online version contains supplementary material available at 10.1007/s12574-022-00568-7.

## Background

Chest pain is one of the most common presenting complaints in the emergency departments and accounts for millions of visits every year [1]. Approximately, 15–20% of these patients have an acute coronary syndrome (ACS) and require admission and treatment [2]. Even in the high-sensitive troponin era a notable number of patients admitted to hospital with chest pain diagnosed with significant coronary artery disease (CAD) do not have elevated troponins [3, 4]. In fact, a study comparing different high-sensitive troponin assays found negative predictive values of troponin to be only 53% (44–62) for detection of significant CAD [5]. Patients presenting in the emergency department with chest pain suggestive of significant CAD remains a diagnostic challenge, meaning that a non-invasive, easily and widely available diagnostic test in addition to troponins to identify or rule out CAD would be highly beneficial.

Measurements of global longitudinal strain (GLS) by speckle tracking echocardiography have shown good results in detecting ischemia, occluded vessels and myocardial necrosis in patients with non-ST-segment elevation myocardial infarction [6]. Normal values of GLS have been reported between − 16 and − 22% [7]. A cutoff GLS value of approximately − 20% have been proposed to rule-out possible significant ischemia in a patient population with suspected non-ST-segment elevation ACS [8]. In a previous review, we argued that GLS has limited accuracy in detecting significant CAD in patients evaluated for chest pain and that the assessment of regional longitudinal strain (RLS) should be studied [9].

In the context of a primary general hospital, we wanted to investigate the sensitivity and specificity of RLS to predict significant CAD in patients without previously known CAD who presented with chest pain suspective of ACS on admission. We hypothesized that assessment of RLS might identify patients with chest pain and negative/slightly elevated troponin in need of an invasive strategy. We also hypothesized that RLS would be more predictive of significant CAD than GLS.

## Methods

### Study population

We consecutively included 150 patients with chest pain suggestive of significant CAD admitted to Vestfold Hospital Trust between February 2013 and March 2015, with an initial troponin T < 30 ng/L (HS-Troponin, Roche) and a normal ECG. Troponin T was measured at admission and 3–6 h later. During the inclusion period, the cutoff value for myocardial damage for the applied assay was changed from > 29 ng/L to > 14 ng/L, according to the new ESC definitions of myocardial infarction [10]. We kept, however, the inclusion criterion of initial troponin < 30 ng/L throughout the study. Maximum troponin was defined as any troponin > 14 ng/L during admission. Before inclusion an independent cardiologist considered the need for invasive coronary angiography (ICA) based on a standard clinical evaluation including history, risk factors, biochemistry. Patients with normal troponin values could be referred for ICA at the discretion of the cardiologist when there was persistent suspicion of significant CAD. We excluded patients < 18 years of age, with a QRS width of > 120 ms, more than moderate heart valve dysfunction, overt heart failure, atrial fibrillation or other continuous arrhythmia, known CAD, and abnormal ECG-findings indicating ischemia. Hypertension was defined as treatment with any antihypertensive drug and hyperlipidemia as treatment with statins on admission. Current smokers had been smoking regularly up to a maximum of 3 months prior to inclusion, and previous smokers had stopped before that time. Family history was defined as premature coronary heart disease in first degree relatives (< 50 years in males and < 55 years in females). In case of more than one first relative with a diagnosis of CAD, an age limit of < 60 years was applied. Diabetes mellitus was registered when the diagnosis was established prior to admission. The regional ethics committee approved the study protocol (2012/1592) and all included patients signed written informed consent.

### Echocardiography

Echocardiography was performed at a median of 18 (IQR 10.5) h after admission using a Vivid E9 scanner (GE Ultrasound, Horten, Norway) and stored digitally. All patients were stabilized and without ongoing chest pain before the echocardiographic examination which was performed in our echocardiographic laboratory or in the observation unit in the emergency department. Left ventricular (LV) ejection fraction (EF) was evaluated by Simpson`s biplane method and cardiac volumes were evaluated as recommended by ASE/EACVI [11]. Patients with regional wall motion abnormalities were not excluded as long as other criteria for inclusion/exclusion were met. Segmental wall motion was assessed in a 16-segment model and scored as normal = 1, hypokinetic = 2, akinetic = 3, and dyskinetic = 4. Wall motion score index was calculated and represents the average value of analyzed segments. Valve dysfunction was defined by the European Society of Cardiology (ESC) guidelines for management of valvular heart disease [12]. LV hypertrophy was diagnosed in the presence of LV mass index > 115 g/m^2^ in men and > 95 g/m^2^ in women measured by M-mode according to the ESC/European Society of Hypertension guidelines [13].

For strain analysis, three consecutive beats in three apical views were recorded. Analyses were performed offline with EchoPac software version 113 and the Q-analysis function provided following the steps suggested by Negishi et al. [14]. Manual editing of the region of interest was performed to evaluate the mid-myocardium throughout the echocardiographic image including the apical region, and without being affected by the mitral valve, aortic root or papillary muscles. The inclusion of the pericardium was avoided. An example is shown in supplemental file 1, Fig. 1. End of systole was defined by the closing of the aortic valve evaluated by Doppler or by visual closure of the valve in the five-chamber view. Peak systolic strain was defined as the point of maximal contraction in systole. Global longitudinal peak systolic strain was calculated directly by the software and referred to as GLS. We excluded images with inadequate tracking of the myocardium where > 1 segment were unavailable for analysis in any of the apical views.

**Fig. 1:**
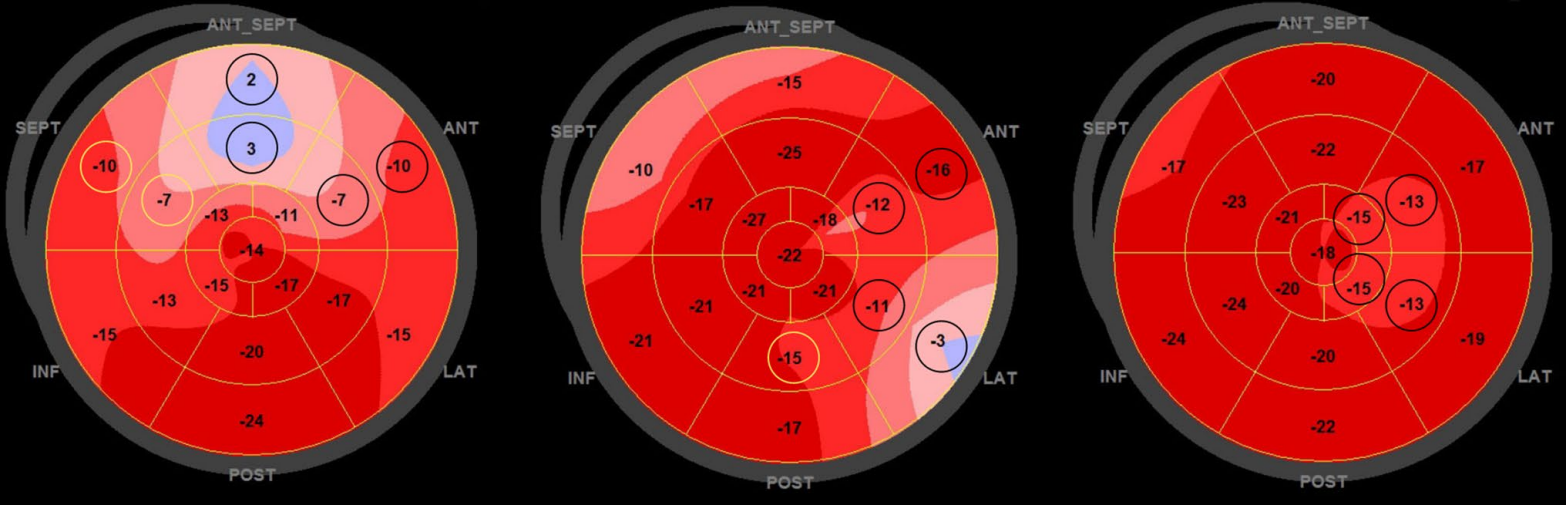
Principles for calculating 4AS. Left Bull’s eye illustrates two alternative ways of calculating 4AS. Alternative 1: two basal segments and two middle segments marked with black circles. Alternative 2: replace the anterior basal and middle segments with the two septal segments marked in yellow which holds the same segment values. The patient had a 100% stenosis. 4AS: − 3.0%. Middle Bull’s eye illustrates calculation of 4AS: two basal and two middle segments marked with black circles are included. The segment marked with a yellow circle is not included, because only two segments from either basal or middle segments can be included in the model for 4AS. The patient had no significant coronary artery stenosis. 4AS: − 10.5%. Right Bull’s eye illustrates calculating 4AS including apical segments: the four adjacent segments are marked in black. The patient had no significant coronary artery stenosis. 4AS: − 14.0%

For the RLS measurements, we arbitrarily selected 10 patients with and 10 patients without significant CAD and evaluated several principles for assessing regional strain without the consideration of coronary perfusion to avoid issues with anatomical variation. We explored three possible models derived from the Bull’s eye presentation (17-segment model) provided by the software based on the concept of Adjacent Segments (AS):

1. 2AS model: The average of the two adjacent segments with the highest values (i.e., least negative) of the middle segments.
2. MidAS: The average of the six middle segments.
3. 4AS model: The average of the four adjacent segments with the highest (least negative) strain values that could be both basal, mid and apical. A maximum of two basal and a maximum of two middle segments out of the four were allowed in this model.

Examples of the 4AS model are presented in Fig. 1. The primary statistical analyses for identifying CAD showed best results for 4AS, and this model was used for further analyses. For 4AS, we excluded images with inadequate tracking in > 1 segment. The operator was blinded to the biomarkers and angiographic data. We used the model of coronary artery perfusion territories proposed by Lang et al. [11] and retrospectively analyzed the association between the location of the 4AS segments in the 17-segment model and the location of the significant stenosis found on the coronary angiogram. Inter- and intraobserver variabilities were evaluated in 20 randomly selected patients. For the interobserver analyses, two observers investigated the same cine-loops blinded to the results of the other. For intraobserver analyses, one observer investigated the same cine-loops approximately 4 weeks apart.

### Coronary angiography

ICA was performed at a tertiary hospital within a median of 3 (IQR 35) days of admission to our hospital. The angiographic and procedural methods were performed and interpreted in accordance with generally accepted guidelines and routines involving digital imaging acquisition and storage. A lesion was deemed significant when it reduced luminal diameter by at least 50% (corresponding to 75% area stenosis) based on visual assessment. Fractional flow reserve, optical coherence tomography, and intravascular ultrasound were not routinely performed.

One experienced invasive cardiologist blinded to the echocardiographic findings reevaluated the available data retrospectively and described the angiograms according to Syntax score [15]. Non-significant CAD patients (CAD−) were characterized as either with minor coronary vessel wall changes (30–49% luminal diameter reduction) or normal coronary arteries (0–29% luminal diameter reduction).

### Statistical analysis

Normality of continuous variables was assessed by visual assessment or Shapiro Wilks test. Homogeneity of variance was tested with the Levene`s test. Continuous data are expressed as mean ± standard deviation (SD) or as median and interquartile range. Categorical variables are reported as numbers and percentages. Comparisons of group means were analyzed using Independent Samples *t* test or Mann–Whitney *U* test for continuous variables and Fisher’s exact test or Chi square test for categorical variables as appropriate. Receiver-operating curve (ROC) analyses were performed using DeLong DeLong, and Clarke-Pearson comparison in MedCalc version 18.9 (MedCalc Software, Ostend Belgium) [16] and the strain value with the highest combination of sensitivity, specificity and the corresponding value was found using the Youden index. Predictive values for GLS and 4AS at different cutoff values were also calculated. We chose GLS values found as normal in previous studies [7]. For 4AS, we chose slightly higher cutoff values because basal and middle segments generally have higher LS values. Multiple logistic regression was used to calculate Odds Ratios and corresponding 95% Confidence Intervals for potential predictors of CAD+. Independent variables in the model were chosen on the basis of clinical relevance and a *p* value < 0.20 in unadjusted analyses. The association between the most significant coronary stenosis found in the ICA and GLS/4AS was tested using simple ordinal logistic regression with categories 0–29%, 30–49%, 50–69%, 70–89%, 90–99% and 100% stenosis. One-way ANOVA analysis was used to assess the difference in mean strain values between patients with one-, two- and three-vessel disease. To evaluate the potential benefit of GLS and 4AS in addition to troponin T, we calculated the event-, non-event and net reclassification improvement when adding different cutoff values of GLS/4AS to a troponin T-value indicative of CAD+/CAD− using MATLAB **(**MATLAB and Statistics Toolbox Release R2019b, The MathWorks, Inc., Natick, Massachusetts, United States). All other statistical analyses were performed using SPSS version 25 (SPSS, Inc, Chicago, IL). Differences were considered statistical significant at the level of two-sided *p* < 0.05. Inter- and intraobserver variabilities were assessed by the intraclass correlation coefficient (ICC).

## Results

### Patient characteristics

**Fig. 2:**
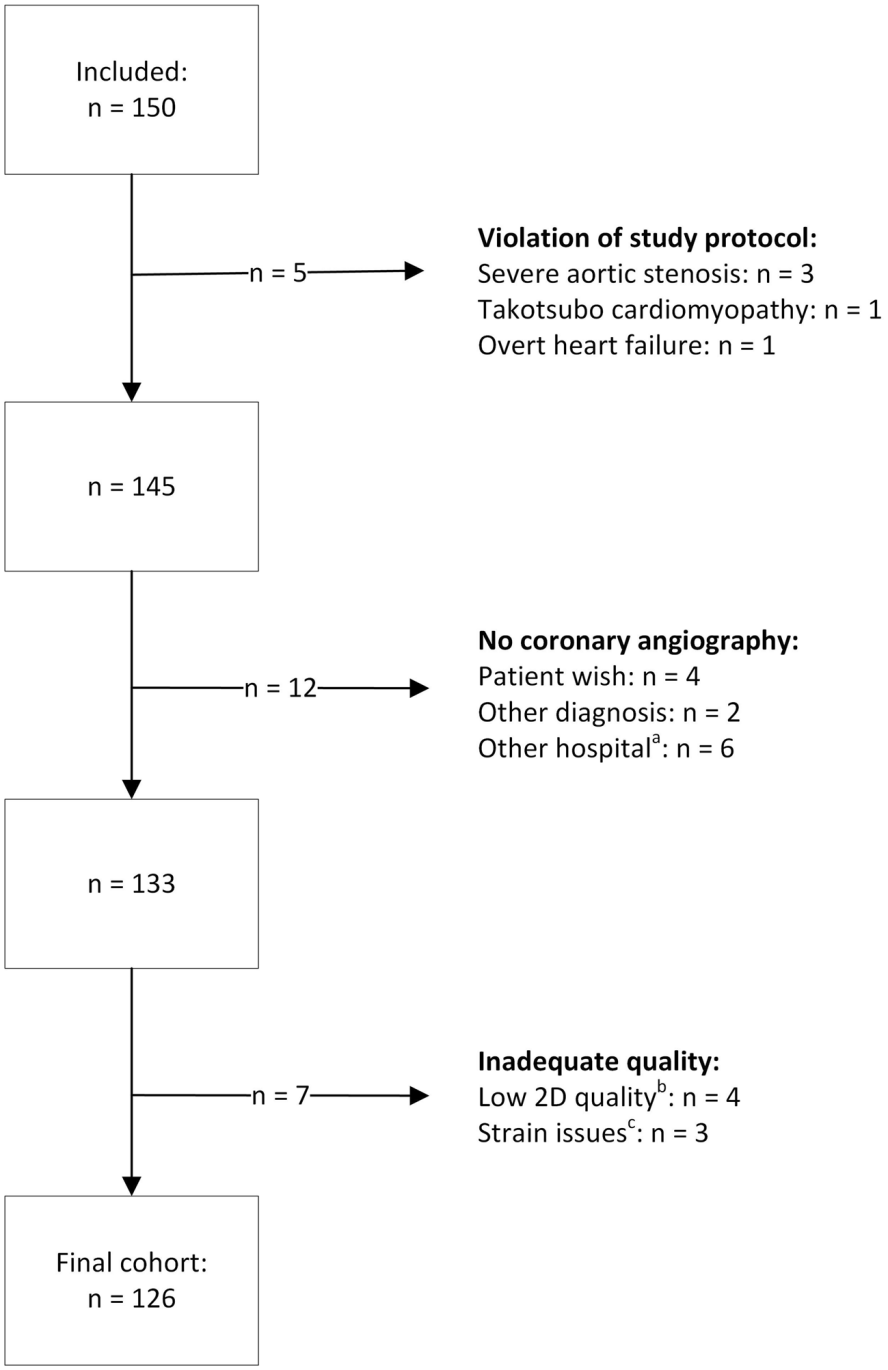
Study population from inclusion to final cohort. *2D* Two-dimensional. ^a^No reports available. ^b^Reverberations, low frame rate. ^c^Changing heart rate

The study flow from all included patients to the final cohort comprising 126 patients is illustrated in Fig. 2. The basic characteristics of the study population subdivided into 37 patients (29%) with CAD+ and 89 (71%) without (CAD−) are summarized in Table 1. CAD+ patients were older, had a higher prevalence of smoking and use of antihypertensive dugs and statins prior to admission than the latter group. They also had a significantly lower proportion with initial troponin *T* ≤ 14 ng/L, a higher percentage with initial troponin *T* 15–30 ng/L and maximum troponin *T* > 14 ng/L at any time (*p* = 0.001 for all). The median time from admission to coronary angiography was 5.5 (IQR 41) days in the CAD− group and 2.0 (IQR 3) days for the CAD+ group (*p* = 0.09). 19/89 patients without significant CAD had at least one troponin value > 14 ng/L. Of these patients, only two had estimated glomerular filtration rate < 60 mL/min/1.73 m^2^. A final diagnosis of type 2 acute myocardial infarction were made in two patients with tachyarrhythmia and coronary artery aneurysms, respectively. The remaining patients were given a final diagnosis of non-coronary chest pain since no obvious cardiac cause for the rise of troponin *T* was found.

**Table 1:** Patient characteristics

	CAD− *n* = 89	CAD+ *n* = 37	*P* value
Age	58 (± 10)	64 (± 12)	0.002*
Male	48 (54)	24 (65)	0.26
Height, cm	174 (± 10)	173 (± 9)	0.59
Weight, kg	80 (± 16)	82 (± 11)	0.62
BMI, kg/m^2^	26.4 (± 3.9)	27.3 (± 3.6)	0.18
Heart rate, bpm	65 (± 12)	67 (± 13)	0.50
Systolic blood pressure, mmHg	132 (± 21)	139 (± 21)	0.10
Diastolic blood pressure, mmHg	78 (± 12)	79 (± 13)	0.47
Risk factors			
Hypertension	27 (30)	18 (49)	0.05
Diabetes	7 (8)	7 (19)	0.07
Hyperlipidemia	17 (19)	10 (27)	0.32
Family history	37 (42)	14 (38)	0.70
Smoker, previous or current	45 (51)^a^	26 (70)	0.049*
Peripheral atherosclerosis	3 (3)	3 (8)	0.36
TIA/stroke	4 (5)	2 (5)	1.00
Medication at enrollment			
Aspirin	17 (19)	12 (32)	0.11
Βeta blockers	9 (10)	4 (11)	0.91
ARB/ACE-I	20 (23)	16 (43)	0.019*
CCB	8 (9)	10 (27)	0.008*
Statins	18 (20)	14 (38)	0.039*
Laboratory results			
Creatinine, mmol/L	70 (± 15)	73 (± 18)	0.36
Hb, g/dL	14.1 (± 1.4)	14.3 (± 1.1)	0.42
Total cholesterol, mmol/L	5.4 (± 1.1)^b^	5.2 (± 1.1)	0.41
Initial troponin T ≤ 14 ng/L	78 (88)	21 (57)	0.000*
Initial troponin T 15—30 ng/L	11 (12)	16 (43)	0.000*
Any troponin > 14 ng/L	19 (21)	19 (51)	0.001*

Continuous variables are presented as mean ± SD and categorical variables as number and percentages unless otherwise indicated

*CAD* coronary artery disease, *BMI* body mass index, *bpm* beats per minute, *BSA* body surface area, *TIA* transient ischemic attack, *ARB* angiotensin receptor blocker, *ACE-1* Angiotensin converting enzyme inhibitor, *CCB* calcium channel blocker, *Hb* hemoglobin

*p value < 0.05

^a^Missing: 1

^b^Missing: 2

### Echocardiography

Conventional echocardiographic findings are summarized in Table 2. The average frame rate was 52 frames/min (± 0.6). Mean LVEF was 55% (± 6.0) and 103 patients (82%) had EF ≥ 50%. Significant differences were found for a higher LV mass index (*p* = 0.001), but not for left ventricular hypertrophy (*p* = 0.21).

**Table 2:** Echocardiographic data

	No CAD *n* = 89	CAD *n* = 37	*P* value
LVEF, %	55 (± 6)	54 (± 5)	0.82
WMSI^a^	1 (0.25)	1 (0.25)	0.13
LVEDD, cm	4.7 (± 0.5)	4.6 (± 0.5)	0.20
LVEDVI, mL/m^2^	69 (± 16)	67 (± 13)	0.53
LVESVI, mL/m^2^	31 (± 9)	30 (± 7)	0.58
LV mass index, g/m^2^	105 (± 26)^b^	124 (± 36)^c^	0.01*
LV hypertrophy *n*, (%)	28 (32)	16 (43)	0.21
LA volume index, mL/m^2^	26 (± 8)	28 (± 12)	0.65

Continuous variables are presented as mean ± SD and categorical variables as numbers and percentages unless otherwise indicated. *LVEF* left ventricular ejection fraction, WMSI wall motion score index, *LVEDD* left ventricular end-diastolic diameter, *LVEDVI* left ventricular end-diastolic volume index, *LVESVI* left ventricular end-systolic volume index, *LA* left atrium

^a^Median and interquartile range *p value < 0.05

^b^Missing: 5

^c^Missing: 4

### Global and regional strain

**Fig. 3:**
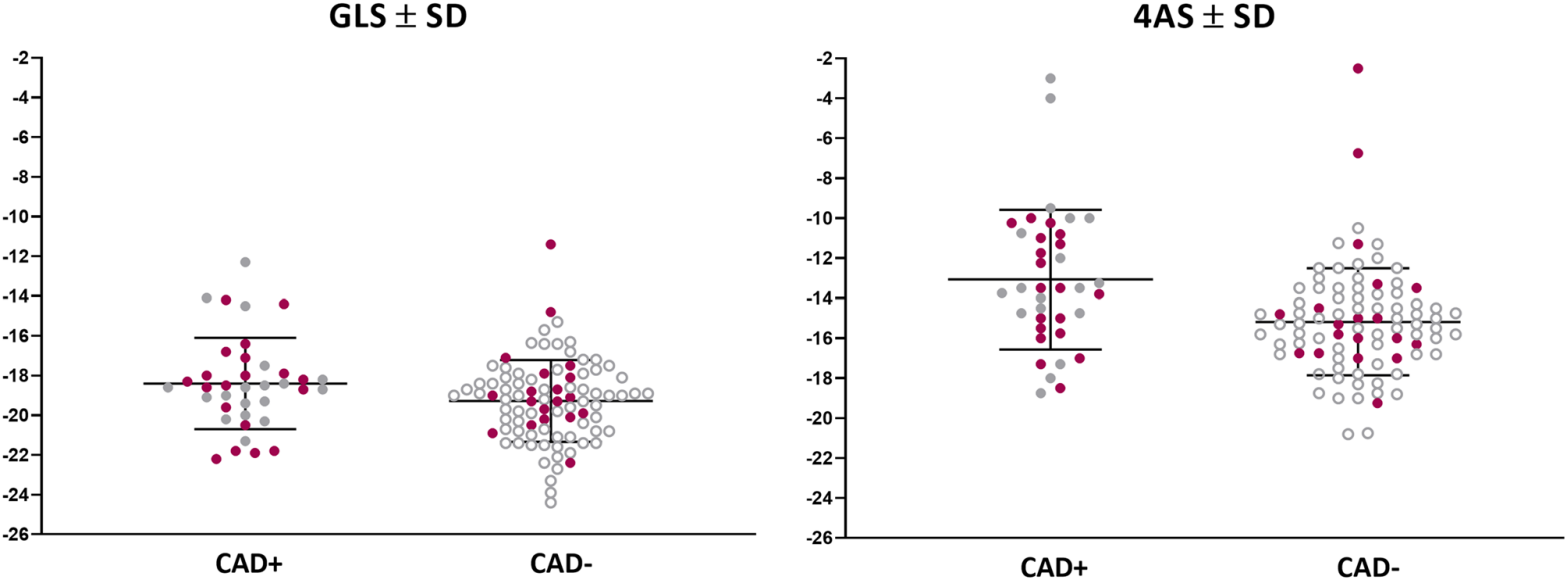
Peak systolic longitudinal strain values ± standard deviations for patients with and without coronary artery disease. *GLS* global longitudinal strain, *SD* standard deviation, *4AS* regional longitudinal strain of the four segments with the highest strain values. Red dots illustrate patients with troponin *T* > 14 ng/L in both CAD+ and CAD− groups

**Fig. 4:**
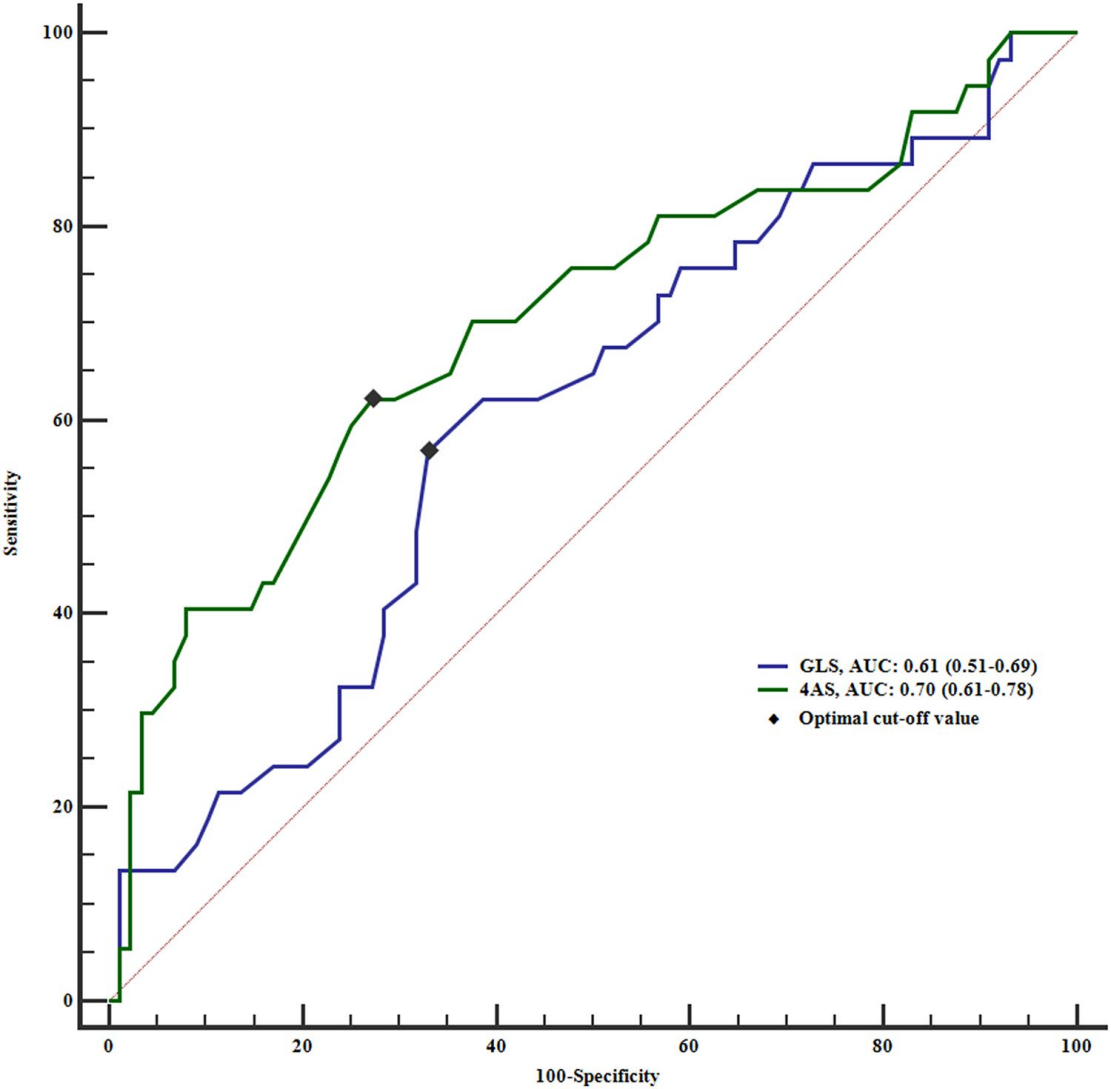
Receiver-operating curve of global longitudinal strain and regional longitudinal strain for detecting significant coronary artery disease. *p* = 0.11 for the difference between curves. GLS, global longitudinal strain; 4AS, regional longitudinal strain of four adjacent segments

Seven patients were excluded due to inadequate speckle tracking (Fig. 2). In one patient (CAD−), GLS measurement was possible, but not 4AS. Individual GLS and 4AS values with and without a maximum troponin *T* > 14 ng/L for both groups are presented in Fig. 3. Mean GLS values were − 18.4% (± 2.3) and − 19.3% (± 2.1) in the CAD+/CAD− group, respectively (*p* = 0.038), independent of troponin level. The respective values for 4AS were − 13.1% (± 3.5) and − 15.2% (± 2.7) (*p* = 0.002). As evident from Fig. 4, the area under curve (AUC) for prediction of CAD+ was 0.70 for 4AS and 0.61 for GLS (*p* = 0.11) for the total cohort with corresponding cutoff values − 14.3% for 4AS and − 18.7% for GLS.

Sensitivity, specificity, AUC and predictive values for GLS and 4AS among patients without any troponin *T* > 14 ng/L at different cutoff values are presented in Table 3. For 4AS, the largest AUC [0.70 (0.59–0.79)] was found for a cutoff value of > − 15%, with a high sensitivity and negative predictive value (NPV), but lower specificity and positive predictive value (PPV). The highest AUC for GLS was 0.62 (0.51–0.72) with a cutoff level of − 20% with intermediate sensitivity and specificity, but lower PPV and a fairly high NPV. The event-, non-event- and net reclassification improvement for 4AS and GLS for different cutoff values are presented in Tables 4 and 5. For 4AS, a cutoff level of − 15% had the best event-reclassification combined with overall positive net reclassification improvement. For GLS, the best event-reclassification was at a cutoff level of − 20%.

**Table 3:** Myocardial function by strain in patients with negative troponin

	Sensitivity (%)	Specificity (%)	PPV (%)	NPV (%)	AUC	*p* value
Strain values						
GLS^a^	94 (73–100)	33 (22–45)	27 (23–31)	96 (77–99)	0.62 (0.51–0.72)	0.076
GLS > − 20%	78 (52–94)	46 (34–58)	27 (21–34)	89 (76–95)	0.62 (0.51–0.72)	0.045*
GLS > − 19%	33 (13–59)	69 (56–80)	21 (12–36)	80 (74–85)	0.51 (0.40–0.62)	0.881
GLS > − 18%	17 (4–41)	87 (77–94)	25 (9–53)	80 (76–84)	0.52 (0.41–0.63)	0.700
4AS^a^	83 (59–96)	61 (48–72)	36 (28–44)	93 (83–98)	0.74 (0.63–0.83)	0.002*
4AS > − 17%	83 (59–96)	23 (14–35)	22 (18–27)	84 (64–94)	0.53 (0.42–0.64)	0.530
4AS > − 16%	83 (59–96)	38 (26–50)	26 (21–32)	90 (75–96)	0.61 (0.49–0.71)	0.051
4AS > − 15%	83 (59–96)	57 (44–68)	33 (26–41)	93 (82–97)	0.70 (0.59–0.79)	0.000*
4AS > − 14%	61 (36–83)	75 (64–85)	39 (27–53)	88 (80–93)	0.68 (0.57–0.78)	0.005*
4AS > − 13%	39 (17–64)	93 (84–98)	58 (34 (80)	85 (80–89)	0.66 (0.55–0.77)	0.010*

Area under curve, sensitivity, specificity and predictive values of strain values and 95% confidence intervals. *GLS* global longitudinal strain, *4AS* regional longitudinal strain average value of the four adjacent segments with the highest values. *PPV* positive predictive value, *NPV* negative predictive value, AUC area under curve

^a^Overall

*p value < 0.05

**Table 4:** Reclassification of patients with and without coronary artery disease using different cutoff values for 4AS in addition to troponin

		4AS^b^ > − 17%			4AS^b^ > − 16%			4AS^b^ > − 15%			4AS ^b^ > − 14%			4AS^b^ > − 13%		
		CAD−	CAD+	Total	CAD−	CAD+	Total	CAD−	CAD+	Total	CAD−	CAD+	Total	CAD−	CAD+	Total
	CAD+ patients															
Troponin^a^	CAD−	3	15^c^	18	3	15^c^	18	3	15^c^	18	7	11^c^	18	11	7^c^	18
	CAD+	0	19	19	0	19	19	0	19	19	0	19	19	0	19	19
	Total	3	34	37	3	34	37	3	34	37	7	30	37	11	26	37
	Event NRI	0.41 (*p* < 0.001)*			0.41 (*p* < 0.001)*			0.41 (*p* < 0.001)*			0.30 (*p* < 0.001)*			0.19 (*p* = 0.008)*		
	CAD− patients															
Troponin^a^	CAD−	16	53^d^	69	26	43^d^	69	39	30^d^	69	52	17^d^	69	64	5^d^	69
	CAD+	0	19	19	0	19	19	0	19	19	0	19	19	0	19	19
	Total	16	72	88	26	62	88	39	49	88	52	36	88	64	24	88
	Non-event NRI	− 0.60 (*p* < 0.001)*			− 0.49 (*p* < 0.0001)*			− 0.34 (*p* < 0.0001)*			− 0.19 (*p* < 0.001)*			− 0.06 (*p* = 0.025)*		
	Overall NRI	− 0.20 (*p* = 0.140)			− 0.08 (*p* = 0.517)			0.07 (*p* = 0.596)			0.10 (*p* = 0.303)			0.13 (*p* = 0.081)		

Net reclassification of events (CAD+), non-events (CAD−) and overall when adding 4AS to troponin as a diagnostic tool. 4AS; regional longitudinal strain average value of the four adjacent segments with the highest values

*CAD* coronary artery disease, *NRI* net reclassification improvement

^a^Classification by troponin

^b^Classification by 4AS in addition to troponin at the respective cutoff value

^c^Number of patients correctly reclassified as CAD+ by 4AS

^d^Number of patients incorrectly reclassified as CAD+ by 4AS

*p value < 0.05

**Table 5:** Reclassification of patients with and without coronary artery disease using different cutoff values for GLS in addition to troponin

		GLS^b^ > − 20%			GLS^b^ > − 19%			GLS^b^ > − 18%		
		CAD−	CAD+	Total	CAD−	CAD+	Total	CAD−	CAD+	Total
	CAD+ patients									
Troponin^a^	CAD−	4	14^c^	18	12	6^c^	18	15	3^c^	18
	CAD+	0	19	19	0	19	19	0	19	19
	Total	4	33	37	12	25	37	15	22	37
	Event NRI	0.38 (*p* < 0.001)*			0.16 (*p* = 0.014)*			0.08 (*p* = 0.083)		
	CAD− patients									
Troponin^a^	CAD−	32	38^d^	70	48	22^d^	70	61	9^d^	70
	CAD+	0	19	19	0	19	19	0	19	19
	Total	32	57	89	48	41	89	61	28	89
	Non-event NRI	− 0.43 (*p* < 0.001)*			− 0.25 (*p* < 0.001)*			− 0.10 (*p* = 0.003)*		
	Overall NRI	− 0.05 (*p* = 0.069)			− 0.09 (*p* = 0.32)			− 0.02 (*p* = 0.73)		

Net reclassification of events (CAD+), non-events (CAD−) and overall when adding GLS to troponin as a diagnostic tool

*GLS* global longitudinal strain, *CAD* coronary artery disease, *NRI* net reclassification improvement

^a^Classification by troponin

^b^Classification by GLS in addition to troponin at the respective cutoff value

^c^Number of patients correctly reclassified as CAD+ by GLS. ^d^Number of patients incorrectly reclassified as CAD+ by GLS

*p value < 0.05

**Fig. 5:**
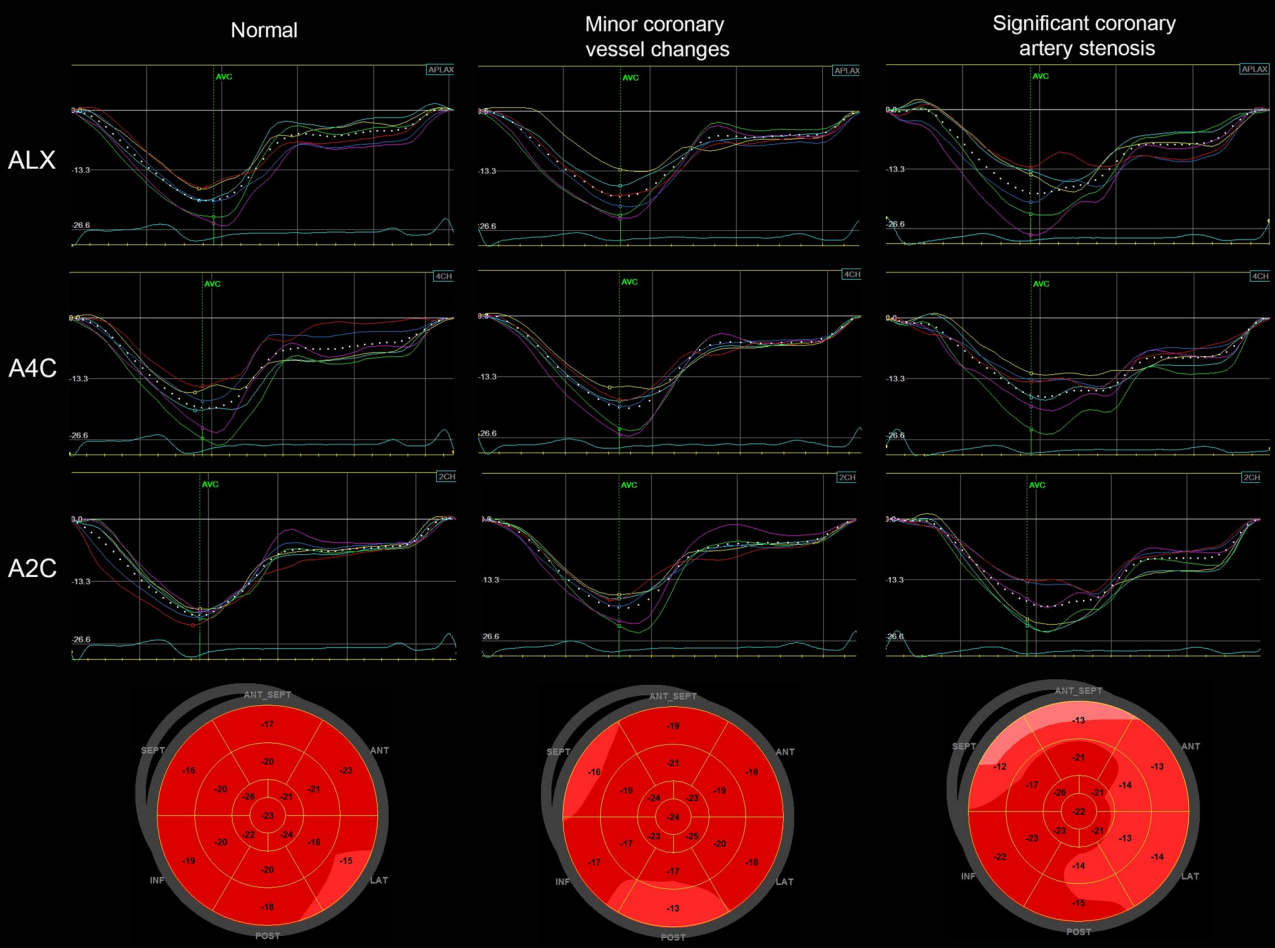
Left column: an example of one patient with normal coronary arteries. GLS: − 20.2%, 4AS: − 17.8%. Middle column: an example of one patient with minor coronary vessel disease—GLS: − 19.7%, 4AS: − 16.0%. Right column: an example of one patient with a significant (70–89%) coronary artery stenosis in the left anterior descending artery. GLS: − 18.5%, 4AS: − 13.5%. *ALX* apical long axis, *A4C* apical four-chamber, *A2C* apical two-chamber, *GLS* global longitudinal strain, *4AS* regional longitudinal strain of four adjacent segments

124 angiograms were available for reanalysis, the remaining two had ICA reports available. Strain curves and Bull’s eye plots from three different patients with normal coronary arteries, minor coronary vessel changes and significant CAD, respectively, are presented in Fig. 5. Coronary angiography findings and strain values related to severity of the disease are presented in supplemental file 1, Table 1. The majority of CAD+ patients (76%) were treated with PCI.

Predictors for significant CAD were age, smoking, maximum troponin *T* > 14 ng/L, and 4AS in adjusted analyses (Table 6). There were no significant difference in the strain values between patients with 1-, 2- and 3-vessel disease (*P* value 0.91 and 0.56 for GLS and 4AS, respectively). Higher (less negative) 4AS values were associated with the degree of the most severe coronary artery stenosis [Odds Ratio 1.21 (1.07–1.38), *p* value 0.002]. This was not significant for GLS [Odds Ratio 1.16 (0.97–1.38), *p* value 0.11]. We were able to do a complete analysis in the Bull’s eye plot from the original investigation in 33/37 patients with significant CAD. In total, 80% of the segments used in the 4AS model were located within the relevant coronary artery territory according to the model for coronary perfusion applied.

**Table 6:** Predictors for CAD+

Variable	Unadjusted OR (95% CI)	*p* value	Adjusted OR (95% CI)	*p* value
Age	1.06 (1.02–1.10)	0.003	1.06 (1.01–1.11)	0.026
Male	1.57 (0.71–3.49)	0.260		
BMI	1.07 (0.97–1.19)	0.182	1.0 (0.87–1.16)	0.978
Family history	0.86 (0.39–1.89)	0.697		
Hypertension	3.55 (1.60–7.91)	0.002	2.48 (0.95–6.47)	0.063
Diabetes	2.73 (0.89–8.45)	0.081	1.66 (0.34–8.27)	0.534
Hyperlipidemia	2.40 (1.03–5.57)	0.041	1.71 (0.59–4.96)	0.325
Smoking*	2.26 (1.00–5.13)	0.051	3.39 (1.21–9.51)	0.020
Troponin > 14 ng/L**	3.89 (1.71–8.83)	0.001	3.32 (1.28–8.60)	0.014
4AS	1.27 (1.09–1.46)	0.002	1.24 (1.05–1.46)	0.010

*OR* Odds Ratio, *4AS*: regional longitudinal strain average value of the four adjacent segments with the highest values

^*^Current and previous

**Maximum value of Troponin measured > 14 ng/L

### Reproducibility

Intra- and interobserver variabilities of 20 patients for GLS were ICC 0.91 (0.77–0.97) and 0.84 (0.57–0.94), respectively. The corresponding values for 4AS were 0.93 (0.83–0.98) and 0.82 (0.49–0.93).

## Discussion

This study demonstrates that assessment of regional myocardial function by strain can be helpful to reveal significant CAD in patients admitted to the emergency department with unexplained chest pain. The inclusion criteria, consisting of normal ECG and first troponin *T* < 30 ng/L, challenge all known diagnostic tools in these patients. Troponin was > 14 ng/L in only 51% of our patients with CAD+, but our findings show that assessment of strain might have incremental diagnostic power also in this group. We also demonstrate that assessment of regional myocardial function by the 4AS method can be useful as a “rule-out” tool in patients with normal troponin levels using ≤ − 15% as a cutoff. Interestingly, we found an association between the degree of the most significant stenosis and the 4AS value, and 4AS was an independent predictor of significant CAD in adjusted analyses. The use of regional strain has been debated due to its relatively poor reproducibility [17, 18]. We suggest, however, that the strategy of averaging the strain value from four adjacent segments with the poorest myocardial function in the Bull’s eye plot is associated with a better diagnostic performance than troponin alone. We were also able to demonstrate good reproducibility for the novel 4AS. Introducing 4AS at a cutoff level of > − 15% increased the number of detected CAD− patients from 51 to 92%. The AUC for both 4AS and GLS was only modest, and the low specificity and negative non-event-reclassification imply that if this method is used, some CAD− patients would still be sent for further investigation (coronary computed tomography angiography or ICA). Several clinical factors such as diabetes mellitus, hypertension and age affect myocardial function. We are not aware of a method for distinguishing the effect of these factors from the effect of significant CAD and the influence of these factors may have contributed to the low specificity of the method. However, theoretically, a method for evaluating regional myocardial function as proposed here could overcome these challenges as they affect global longitudinal function. Keeping in mind the limited number of patients with significant CAD, the finding that 4AS was independently associated with significant CAD adjusted for hypertension and diabetes supports this theory.

All clinical, echocardiographic and ICA data were prospectively collected and analyzed by experienced examiners according to recent guidelines, with reexamination of 98% of ICAs by one highly experienced invasive cardiologist.

Several studies have reported both GLS and RLS to be sensitive in detecting CAD, but there are lack of studies combining troponin and myocardial strain. One study defined RLS as two or more adjacent segments within the same level (basal, mid or apical) and tested different cutoff ranging from − 11 to − 15%. RLS had better diagnostic performance than GLS, similar to our study. [19]. In a smaller study, Smedsrud et al. [20] investigated patients referred to elective ICA for suspected CAD. Both GLS and territorial RLS showed moderate sensitivity/specificity for predicting CAD.

A multi-center study by Shiran et al. [21] concluded that neither GLS nor the 20% worst strain values were useful tools to rule out ACS in the emergency department.

Compared to other studies, the difference in GLS between CAD+ and CAD− was barely significant and this may be related to the inclusion of a more diverse group of patients without clear clinical evidence of CAD+ on admission. In addition, a dilution effect of patients with CAD− who had a rise of troponins related to myocardial damage, but without evidence of angiographic CAD may have been present.

Even so, the difference observed with 4AS was more pronounced and underlines the value of using a regional variable to detect regional myocardial disease, especially in those patients with a normal troponin. The difference of the diagnostic performance of GLS and 4AS may be due to both technical and clinical factors. GLS is calculated using the mean values over the entire length of the myocardial wall and is not calculated from the average of the 17 segments presented in the Bull’s eye. Compensatory hypercontraction and the susceptibility of the apical segments to gain more negative values may be other explanatory factors. In addition, as previously mentioned, GLS may be more vulnerable to the influence of other clinical factors as hypertension, age and diabetes.

Different methods for assessing regional strain have been suggested in previous studies [6, 19, 22]. Regional measurements as 4AS tested here may be more appropriate than GLS in this population, but the optimal method for regional strain assessment is still unknown. Currently, an automated assessment of 4AS is not commercially available. However, such an automated tool could easily be developed to facilitate clinical availability if the method proves clinically valuable in future studies.

### Limitations

The sample size is small, and only 37 patients had significant CAD. Therefore, the results of the study may not represent all patients with unexplained chest pain on admission, and our findings will need to be confirmed in larger studies. The inclusion of a minority of patients with slightly elevated initial troponin T to the level of 14–30 ng/L after initiation of the study may be regarded as a limitation that may also have represented selection bias. An independent cardiologist selected the patients for ICA, but no record was kept of the patients who were not selected for ICA and may also represent a source for selection bias. Although 51% of CAD+ patients had elevated troponin T prior to angiography, an interesting finding of this study was that this was also observed in one-fifth of CAD− patients. If more stringent inclusion criteria, such as initial troponin T < 14 ng/L had been applied, we would have had less CAD− patients with elevated maximal troponin T, but as seen in the present study such a minor elevation was not necessarily associated with significant, angiographically verified CAD. One could argue that although the elevated troponin T levels in CAD− patients did not represent significant CAD, the myocardial necrosis may have influenced strain results. Performing echocardiography with regional strain analysis requires an experienced cardiologist/echocardiographer, good quality images and additional time for subsequent assessment of regional strain. These demands might be an important limitation for its routine use in different acute clinical settings.

## Conclusions

A novel 4AS approach to evaluate decreased myocardial function in four adjacent LV segments assessed by strain has the potential to detect significant CAD in patients admitted to the emergency department with chest pain suspective of ACS. In patients with normal troponin levels, a 4AS value of ≤ − 15% may be helpful as a “rule out” tool. This strategy had better diagnostic qualities than GLS in this population and seems to overcome the problems reported for reproducibility for regional strain analyses.

## Supplementary Information

Below is the link to the electronic supplementary material.

Supplementary file1 (DOCX 67 KB)
