# Supplementary material for: Novel regional longitudinal strain by speckle tracking to detect significant coronary artery disease in patients admitted to the emergency department for chest pain suggestive of acute coronary syndrome
Source: J Echocardiogr. 2022 Mar 15;20(3):166–77. doi: 10.1007/s12574-022-00568-7 (PMC9374627; doi:10.1007/s12574-022-00568-7)
Supplement: Supplementary file 1 — Supplementary file1 (DOCX 67 KB) [file 12574_2022_568_MOESM1_ESM.docx]

**Supplementary information**

**Figure 1**

Title: Region of interest


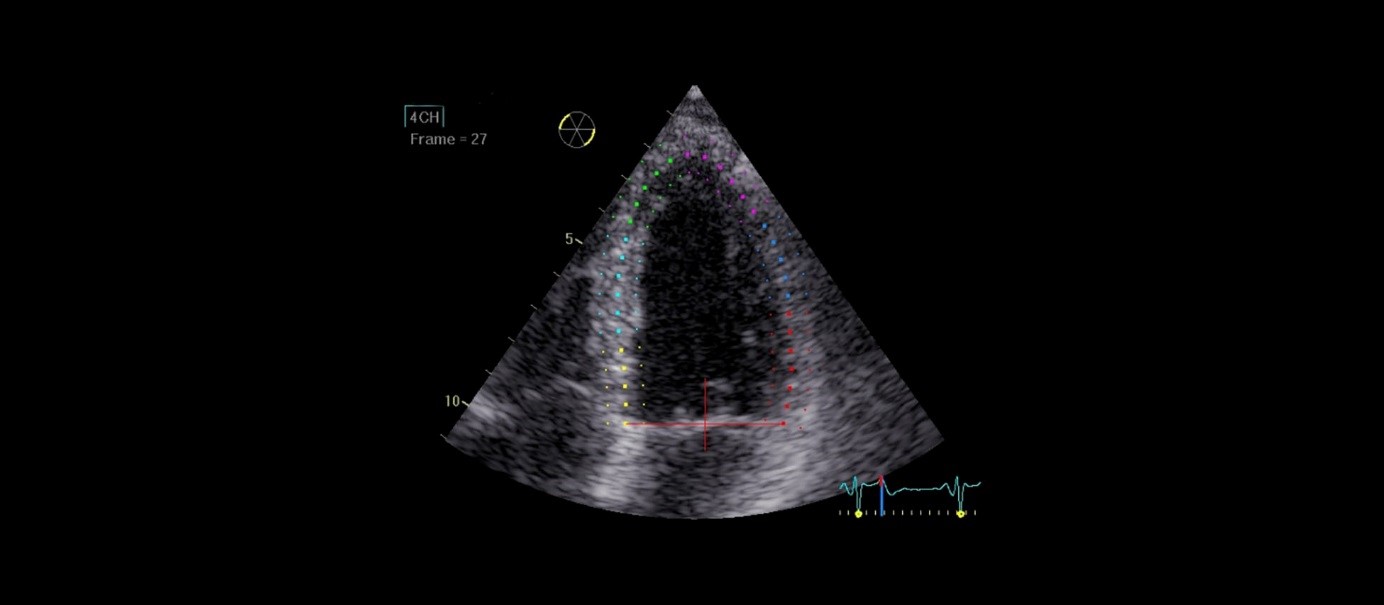


Legend: Illustration of drawing of the region of interest where the middle dotted line covers the mid-myocardium.

**Table 1**

Title: Coronary angiography findings and strain values related to severity of the disease.

| **Results of coronary angiography** | **n** | **GLS %** | **4AS %** |
| --- | --- | --- | --- |
| **Normal (0-29% stenosis)** | 80 | -19.2 (± 2.1) | -15.2 (± 2.8) |
| **Minor coronary vessel changes (30-49% stenosis)** | 9 | -20.0 (±1.8) | -15.4 (± 2.0) |
| **Significant coronary artery disease (≥ 50% stenosis)** | 37 |  |  |
| 1-vessel disease | 23 | -18.2 (± 2.0) | -13.0 (± 3.2) |
| 2-vessel disease | 8 | -18.1 (± 3.0) | -11.5 (± 4.6) |
| 3-vessel disease | 6 | -18.3 (± 2.9) | -13.3 (± 3.2) |
| Main stem | 2 |  |  |
| RCA | 21 |  |  |
| LAD | 20 |  |  |
| CX | 8 |  |  |
| **Number of lesions*** |  |  |  |
| 1 | 17 |  |  |
| 2 | 8 |  |  |
| 3 | 3 |  |  |
| 4 | 4 |  |  |
| 5 | 1 |  |  |
| 6 | 2 |  |  |
| **Treatment**  PCI  CABG  Conservative |  |  |  |
|  | 28 |  |  |
|  | 2 |  |  |
|  | 7 |  |  |

Legend: Summary of findings on coronary angiography. RCA: Right coronary artery, LAD: Left anterior descending, CX: Circumflex, PCI: Percutaneous coronary intervention, CABG: Coronary artery bypass graft, GLS: Global longitudinal strain, 4AS: Regional longitudinal strain average value of the four adjacent segments with the highest values.*Detailed information on lesions not available in 2 participants.
